# Origin of fecal contamination in lettuce and strawberries: From microbial indicators, molecular markers, and *H.pylori*

**DOI:** 10.1016/j.heliyon.2024.e36526

**Published:** 2024-08-19

**Authors:** Fidson-Juarismy Vesga, Camilo Venegas, Valentina Flórez Martinez, Andrea C. Sánchez-Alfonso, Alba Alicia Trespalacios

**Affiliations:** a Microbiology Department, Grupo de Biotecnología ambiental e industrial (GBAI), Laboratorio Calidad Microbiológica de Aguas y Lodos (CMAL), Science Faculty, Pontificia Universidad Javeriana, Bogotá, Colombia. Carrera 7 No. 43 - 82, Bogotá, 110231, Colombia; b Corporación Autónoma Regional de Cundinamarca, Avenida Calle 24 (Esperanza) # 60 - 50, Centro Empresarial Gran Estación, Costado Esfera Pisos 6-7, Bogotá, 111321, Colombia; c Microbiology Department, Infectious Diseases Research Group, Science Faculty, Pontificia Universidad Javeriana, Bogotá, Colombia, Carrera 7 No. 43-82, Bogotá, 110231, Colombia, Bogotá, Colombia

**Keywords:** Fecal pollution, Food safety, Food-borne pathogen, Indicator bacteria, Microbial source tracking

## Abstract

Around 2 billion people utilize a water source contaminated with fecal-origin microorganisms, used for both human consumption and irrigation of crops. In Colombia, the water from the Bogotá River is employed for irrigating agricultural products, including raw-consumption foods like strawberries and lettuce. This poses a risk to the end consumer, as these foods are marketed as fresh products ready for direct consumption without undergoing any disinfection or cooking treatment. The aim of this study was to determine the origin of fecal contamination in strawberries and lettuce irrigated with surface waters from Cundinamarca, Colombia, using non-human and human molecular markers, along with *Helicobacter pylori* (*H. pylori)*. A total of 50 samples were collected, 25 of strawberries and 25 of lettuce, taken from crops, markets, and supermarkets. Microbiological indicators (bacterial and viral) were detected through cultivation techniques, and Microbial Source Tracking (MST) markers and *H. pylori* were detected through PCR. The results of our study demonstrate the presence of *Escherichia coli (E. coli)* (12.5 %), Enterococcus (≥25 %), spores and vegetative forms of *Spores of sulphite-reducing Clostridia (SRC)* (≥37.5 %), coliphages (*≥*12.5 %), and *Salmonella* sp. (≥12.5 %), in both strawberries and lettuce. In the different samples analyzed, molecular markers were detected to differentiate the source of fecal contamination above 12.5 % (HF187, CF128, ADO and DEN) and *H. pylori* between 0 % and 25 %, highlighting deficiencies in the production chain. of food, and the risks they pose to food security. Highlighting deficiencies in the food production chain and the risks they pose to food safety.

## 1 Introduction

Waters from contaminated sources are extensively reused, particularly in developing countries. It's estimated that approximately 2 billion individuals rely on water contaminated with fecal microorganisms for various purposes, including human consumption and crop irrigation [1]. This practice is prevalent in the cultivation of raw consumption foods like strawberries and lettuce, which demand substantial water volumes [2,3]. Consequently, nearby water sources are often utilized without regard for their quality. In many instances, the water quality is subpar, rendering it unsuitable for agricultural purposes [[4], [5], [6]]. Despite farmers' awareness of the risks posed to their crops [5,7,8], such water is indiscriminately employed. In Colombia, for example, water from the Bogotá River is used for agricultural irrigation [9], despite its contamination levels ranging from acceptable to moderate [10]. In the upper and middle basin of the Bogotá River, a staggering 97 % of water is allocated for irrigating crops such as strawberries, vegetables, potatoes, and grass [11,12]. This presents a health hazard as some of these foods are marketed as fresh products intended for direct consumption without any disinfection treatment [13,14], contrary to Codex Alimentarius guidelines [4].

Identifying and tracing the origin of microbiological contamination in water and food, along with detecting pathogenic microorganisms responsible for foodborne illnesses, represent significant challenges. Such monitoring serves as a vital tool for food regulatory bodies and health and environmental authorities, enabling them to understand and control contamination sources through preventive measures, transmission mitigation, or the permanent elimination of fecal contamination sources in water and food [15,16]. However, in most cases, this pursuit and monitoring are lacking, with only traditional indicator microorganisms, typically bacteria like *Escherichia coli* (*E. coli*), being assessed. This is done either to evaluate inadequate hygiene practices or as indicators of food production process failures [14].

While the detection of traditional indicator microorganisms and, occasionally, certain pathogens is commonplace, enteric viruses gain significance due to their high incidence and associated outbreaks linked to agricultural product consumption [17]. These viruses have been detected in various contaminated irrigation waters [18,19], with their presence often associated with food handling issues [17]. However, assessing viruses poses challenges due to the complexity and high costs of detection techniques. Consequently, the use of viral indicators, such as RNA-specific phages, emerges as an alternative due to their ease of detection and association with enteric viruses [[20], [21], [22], [23]].

Furthermore, identifying sources of fecal-origin microorganism contamination in fresh products is challenging due to their short shelf life, distribution logistics, and rapid consumption, leading to a loss of traceability and evidence. Similarly, the microbiological contamination route of foods poses concerns, as it can occur via various pathways, including the addition of biosolids to soil, the use of contaminated water resources, improper handling of fresh produce, or the presence of domestic animals in cultivation areas [4,24].

To differentiate fecal contamination sources in foods, Microbial Source Tracking (MST) markers, such as Bacteroides, provide valuable insights [25]. Their discrimination capacity improves when multiple MST markers are used in combination, allowing for the association of their presence with specific contamination sources in different water types [[26], [27], [28], [29]]. Although primarily evaluated in water sources, some studies have utilized multiple markers to trace contamination sources in foods, providing crucial information [25,30,31]. MST markers have proven instrumental in tracing pathogenic strains associated with outbreaks, from infected individuals to contaminated foods, spanning various stages from production to consumption [15,28].

Given the importance of differentiating the source of fecal microbiological contamination in water and food, assessing additional indicators that complement traditional microbiological indicators and detect pathogens like *Helicobacter pylori* (*H. pylori)* is essential [[32], [33], [34], [35]]. Therefore, evaluating specific molecular markers of human origin as substitutes for directly detecting pathogens becomes imperative.

The aim of this study was to determine the source of fecal contamination in strawberries and lettuce irrigated with surface waters in Cundinamarca, Colombia, using both non-human and human molecular markers, including *H. pylori*.

## 2 Materials and methods

### 2.1 Sampling

**Fig. 1:**
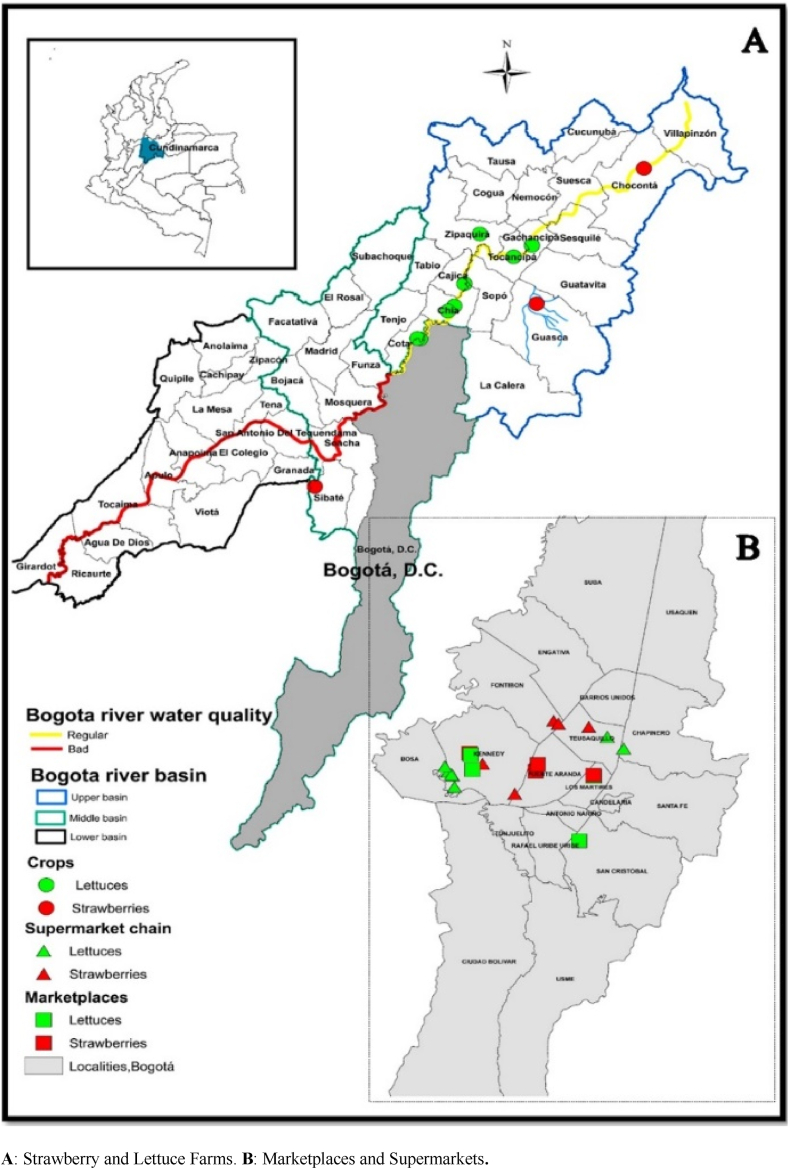
Location of the study site and sampling sites for strawberries and lettuce. **A**: Strawberry and Lettuce Farms. **B**: Marketplaces and Supermarkets.

A total of 50 samples were collected, with 25 corresponding to strawberries and 25 to lettuce. Of these, eight samples were taken from farms (Fig. 1A), eight from marketplaces, and nine from supermarkets (Fig. 1B). The strawberry samples were sourced from farms located in the municipalities of Chocontá, Guasca, and Sibaté (Cundinamarca, Colombia), which are irrigated with waters from the Bogotá River and its tributaries, such as the Chimisé and La Vieja streams. Meanwhile, the lettuce crops were located in the municipalities of Chía, Cota, Cajicá, and Zipaquirá (Cundinamarca, Colombia) (Fig. 1A).

Regarding the commercial samples, they were randomly collected from the main marketplaces and supermarkets in Bogotá, Colombia. These locations are distributed across different districts of the city (Fig. 1B). Following collection, the samples were transported to the laboratory and refrigerated at 4 (±2) °C [36].

### 2.2 Preprocessing of strawberry and lettuce samples for bacteria detection

The preprocessing was conducted following the ISO 6887–1:2017 method [37]. Fifty grams of sample were inoculated into 450 mL of peptone water. For strawberry samples, a concentration of 2X was used, while for lettuce samples, a concentration of 1X was employed. Subsequently, homogenization was performed using an orbital shaker (Barnstead Lab-Line, USA) for 1 h at 250 rpm at room temperature to facilitate analysis and filtration procedures. The samples were then transferred to Whirl-Pak filter bags (Nasco, USA).

### 2.3 Microbiological indicators

#### 2.3.1 Total coliforms and *Escherichia coli*

The quantification of total coliforms (TC) and *E. coli* (Colony Forming Units, CFU, per gram) was conducted following the ISO 9308-1 method [38]. Sample filtration was performed using 0.45 μm × 47 mm cellulose acetate membranes (Sartorious Minisart Biotech, Germany) and a vacuum filtration system (Sartorious Minisart Biotech, Germany). The membranes were placed onto Chromocult agar (Merck, Germany), and the plates were then incubated at 37 (±2) °C. The presence of colonies on Chromocult agar exhibiting dark blue/violet coloration was counted as *E. coli*, and the sum of red colonies and *E. coli* colonies was enumerated as TC. *E. coli* ATCC 25992 and *Klebsiella pneumoniae* ATCC 700603 were used as positive controls, while *Salmonella enterica* ATCC 13076 was used as a negative control.

#### 2.3.2 Enterococcus

Quantification of fecal Enterococcus was conducted following the SM 9230C procedure [39]. Membrane filtration was performed, and the membrane was then placed onto Enterococcus agar according to Slantetz and Bartley (Merck, Germany) and incubated for 48 (±4) hours at 35 (±0.5) °C. Subsequently, a confirmatory test was conducted on Bile Esculin Azide (BEA) agar (Merck, Germany), and these plates were incubated for 2 h at 44 (±0.5) °C. The number of *Enterococcus* (*E. faecalis)* colonies was reported as CFU/g. *E. faecalis* ATCC 1933 was used as a positive control, and *E. coli* ATCC 25922 was used as a negative control.

### 2.4 Spores of sulphite-reducing Clostridia

For the quantification of *Clostridium* spp. sulfite reducers (SRC), the ISO 6461–1 [40] method was followed. The samples were pre-enriched in buffered peptone water, which was previously treated at 75 °C for 15 min to transition the bacteria from the vegetative to the spore form. In parallel, samples without heat treatment were analyzed to preserve the vegetative form of the bacteria. Subsequently, filtration was performed using 0.22 μm × 47 mm nitrocellulose membranes. These membranes were placed onto SPS agar (Merck, Germany), and 2–5 mL of Sulfite Polymyxin Sulfadiazine Agar (SPS) (Oxoid, UK) at a temperature between 45 and 60 °C were added to each plate, covering the entire membrane to create an anaerobic environment.

The plates were incubated under anaerobic conditions using an AnaeroGen™ sachet (Oxoid, UK) for 44 (±4) hours at 37 (±2) °C. *Clostridium* sp. CMPUJ 262 was used as the positive control, and *E. coli* ATCC 25922 was used as the negative control. The result was reported as *Clostridium* sulfite reducer spores (SSRC) as CFU/g of the analyzed sample. For the sample pre-treated in buffered peptone water without heating under the same aforementioned conditions, it was reported as CFU/g of *Clostridium*.

### *2.5* *Salmonella* spp.

The quantification of *Salmonella* spp. was conducted following the ISO 6579–1:2017 method [41]. This method involves inoculating the sample into 3 series of 5 tubes of Tryptic Soy Broth (TSB) medium (Merck, USA). For subsequent identification, specific selective media for *Salmonella*, such as Modified Semi-Solid Rappaport-Vassiliadis (MSRV) (Oxoid, UK) and Xylose Lysine Deoxycarboxylase (XLD) agar (Oxoid, UK), were used, followed by confirmation through biochemical tests. *Salmonella enteritidis* ATCC 13076 was used as the positive control, and *E. coli* ATCC 25922 was used as the negative control. The result was reported as Most Probable Number (MPN) of *Salmonella*/50 g of analyzed sample.

### 2.6 Pre-treatment of strawberry and lettuce samples for the detection of viral indicators

The pretreatment involved placing 50 g of food sample, either lettuce or strawberries, in 200 mL of elution buffer (100 mM Tris-HCl, 50 mM glycine, and 3 % beef extract, pH 9.5) supplemented with 0.5 M MgCl_2_ [42,43]. Subsequently, the solution was agitated (Barnstead Lab-Line, USA) at 250 rpm for 1 h at room temperature. Whirl-Pak filter bags (Nasco, USA) were used to facilitate the filtration and concentration process.

#### 2.6.1 Enumeration of CB390 and F-RNA bacteriophages

From the resulting elution solutions as described above, 100 mL were filtered through a 0.22 × 47 mm acetate-nitrate cellulose membrane (Sartorious Misart Biotech, Germany). This membrane was then cut into eight fragments and placed in a glass flask containing 5 mL of elution solution (1 % beef extract, 0.05 mol L-1 NaCl, and 3 % Tween 80). Subsequently, the flask was placed in a sonicator (Elma E30H, Germany) for 4 min, and the eluted bacteriophages were quantified by infecting the host strain *E. coli* CB390 (CECT9198) [44]. For the detection of specific RNA phages, the bacterium *Salmonella* Typhimurium WG49 (ATCC 700730) was used as described in ISO10705-1 [45]. Bacteriophages that formed plaques due to the infection of the host strain *S*. Typhimurium WG49 were counted as total F-phages, and the difference between the total and the number of plaques counted in plates with 40 μg/mL RNase in the assay medium was attributed to specific F-RNA phages. The results were expressed as Plaque Forming Units (PFU) per gram.

### 2.7 Detection of discrimination markers of the origin of fecal contamination

#### 2.7.1 DNA extraction from strawberries and lettuce

From the pre-treated samples, 200 mL of each sample were taken and centrifuged at 3000×*g* for 20 min. The resulting pellet was resuspended in 2 mL of phosphate-buffered saline (PBS 1 × : 130 mmol/L sodium chloride, 10 mmol/L sodium phosphate, pH 7.2). DNA was purified from a 1 mL aliquot of each concentrated sample using the DNeasy Blood & Tissue kit (Qiagen, USA), following the manufacturer's instructions. The DNA was stored at −20 °C (±5 °C).

#### 2.7.2 Detection of Bacteroidetes group

To determine the presence of Bacteroidetes, a specific primer set was used to discriminate between human fecal contamination (HF183) and ruminant fecal contamination (CF128). The use of a CF128 and HF183 marker specific to ruminant sources was essential due to the predominance of livestock activities in the Bogota River region. Additionally, the Bogota River receives wastewater from metropolitan areas and small towns and small slaughterhouses. Fragments of 520 bp and 580 bp, respectively (HF183: 5′ATCATGAGTTCACATGTCCG3′ and CF128F: 5′CCAACYTTCCCGWTACTC 3′, and reverse Bac708R: 5′CAATCGGAGTTCTTCGTG 3′) were amplified [46]. Amplification was performed using the GoTaq® Green Master Mix (Promega, M7123, USA) commercial mix. The final reaction volume was 10 μL, containing 1U of GoTaq Green 2X (Promega, USA), 0.5 μL of each primer (10 mM), and 1 μL of template DNA. *Bacteroides fragilis* RYC 2056 (ATCC 700786) DNA was used as a positive control for ruminant fecal contamination, and the strain *B. thetaiotaomicron* GA17 was used for human fecal contamination.

The amplification conditions were as follows: initial denaturation of DNA at 94 °C for 2 min, followed by 35 cycles of 94 °C for 1 min, 62 °C for 1 min for the ruminant marker, 63 °C for 1 min for the human marker, and 72 °C for 1.5 min, followed by a final extension at 72 °C for 7 min [29]. The reaction mixture was kept at 12 °C (T10 Thermal Cycler, BIO-RAD, USA). All samples and controls were run in duplicate.

#### 2.7.3 *Bifidobacterium* group detection

For the detection of the *Bifidobacterium* group, a nested PCR was performed. Initially, amplification was carried out using specific primers for the *Bifidobacterium* genus, Lm26 (5′GATTCTGGCTCAGGATGAACG 3′) forward and Lm3 (5′CGGGTGCTICCCACTTTCATG) reverse [47], resulting in a 1.35 kb fragment. Subsequently, an ADO-DEN multiplex PCR was performed to detect *Bifidobacterium adolescentis* (BI-ADO-1) (5′CTCCAGTTGGATGCATGTC3′), BI-ADO-2 (5′CGAAGGCTTGCTCCCAGT3′), and *Bifidobacterium dentium* (BI-DEN-1) (5′ATCCCGGGGGTTGCGCT 3′), (BI-DEN-2) (5′GAAGGGCTTGCTCCCGA 3′) [48].

In the PCR analyses, the final volume for each reaction was 10 μL, containing 5 μL of GoTaq® Green Master Mix (Promega, M7123, USA), 0.5 μL of each primer (10 mM), and 2 μL of DNA template. The amplification was performed using the T10 Thermal Cycler (BIO-RAD, USA) under the conditions described by Matsuki et al., 1999 [48]. *Bifidobacterium adolescentis (B. adolescentis)* DSM 20083 and *Bifidobacterium dentium (B. dentium)* DSM 20084 were used as positive controls.

#### 2.7.4 Detection of DNA *Helicobacter pylori*

The detection of *H. pylori* was performed by conventional PCR amplification of the *vac*A gene using the primers sequences proposed by Nilsson et al. [49] (F: 5′-GGCACA CTG GAT TTG TGG CA -3′ and R: 5′-CGCTCG CTT GATTGG ACA GA -3′), amplifying a 372 bp fragment. The amplification conditions used were those established by Vesga F.J et al. [35]. The specificity of the primers was verified in silico using the NCBI database (www.ncbi.nlm.nih.gov) and the BLAST program (www.ncbi.nlm.nih.gov/BLAST), by amplifying DNA from *H. pylori* reference strains NCTC 11637 and 11638 and *E. coli* ATCC 25922.

In the PCR assays, the final reaction volume of 10 μL contained 5 μL of GoTaq® Green Master Mix (Promega, M7123), 0.5 μL of each primer (10 mM), and 1 μL of DNA template. A positive control with *H. pylori* DNA strain NCTC 11637 and a control of external contamination consisting of PCR mix without DNA were included in each PCR analysis, and *E. coli* DNA strain ATCC 25992 was used as a negative control. All food samples and controls were run in triplicate. The PCR products were analyzed by agarose gel electrophoresis using 2 % agarose gel in 1X TAE buffer (Tris-Acetic-EDTA), stained with 0.02 % SYBR® Safe–DNA gel stain (Invitrogen, USA); electrophoresis was carried out at 80 V for 60 min. After completion, the presence of amplified fragments was visualized using the Gel DocTM XR + Imaging System Molecular Imager (BIO-RAD, USA).

#### 2.7.5 Analysis

The data was processed in Excel to identify averages, maximum and minimum values, and frequency indices represented as percentages. The graphs were created using the GraphPad Prism 8 software.

## 3 Results

### 3.1 Microbiological indicators in strawberries and lettuces

**Fig. 2:**
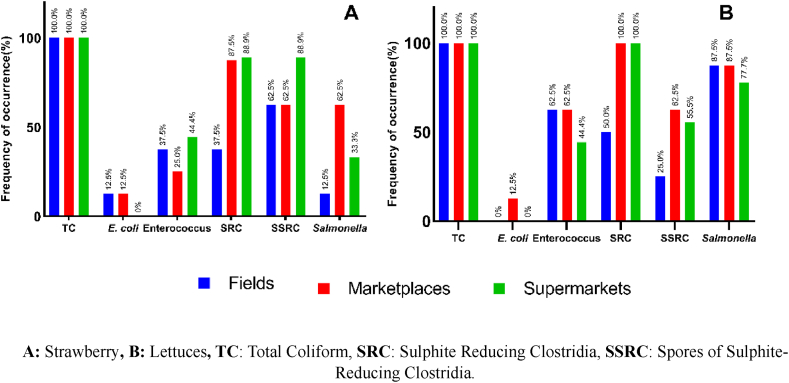
Frequency of appearance of indicator and pathogenic microorganisms in samples of strawberries and lettuce. **A:** Strawberry**, B:** Lettuces**, TC**: Total Coliform, **SRC**: Sulphite Reducing Clostridia, **SSRC**: Spores of Sulphite-Reducing Clostridia.

A total of fifty (50) samples were analyzed (25 strawberries and 25 lettuce), eight (8) were taken from fields, eight (8) from marketplaces, and nine (9) from chain markets. In all strawberry samples, the presence of total coliforms was reported; however, *E. coli* was found in one sample from fields (1/8) and one obtained from marketplaces (1/8), with a prevalence of 12.5 % (1/8) in both cases (Fig. 2). The maximum concentrations of this fecal indicator in field and marketplace samples were 5.7 and 5.30 (Log_10_ CFU/g), respectively (Table 1). Additionally, Enterococcus was predominantly found in samples from supermarkets (44.4 %; 4/9) followed by fields (37.5 %; 3/8), with average concentrations of 1.1 and 1.2 (Log_10_ CFU/g), respectively. Notably, strawberries from fields exhibited maximum concentrations of 4.3 (Log_10_ CFU/g). Regarding strawberries obtained from marketplaces (25 %; 2/8), they presented a lower average concentration (0.8 Log_10_ CFU/g) but with a maximum of 3.6 (Log_10_ CFU/g) (Fig. 2 and Table 1).

**Table 1:** Concentrations of microbiological and viral indicators in the different samples of strawberries and lettuce.

Matrix	Origin	TC Lo_g10_ (UFC/g)	*E. coli* Log_10_ (UFC/g)	EnterococcusLog_10_ (UFC/g)	*SRC*Log_10_ (UFC/g)	SSRCLog_10_ (UFC/g)	*Salmonella* spp. *NMP/50g*
Strawberry (n:25)	Fields (n:8)	5.3(4.3–6.1)	0.7(<0.6–5.7)	1.2 (<0.6–4.3)	1(<0.6–1.4)	0.9 (<0.6–1.8)	<0.006 (<0.006–0.007)
	Marketplace (n:8)	5.9 (5.3–6.5)	0.7(<0.6–5.3)	0.8 (<0.6–3.6)	1.1 (0.9–1.6)	0.7(<0.6–1.6)	0.008 (<0.006–0.014)
	Supermarket (n:9)	5.6(4.8–7.2)	<0.6(<0.6 - <0.6)	1.1(<0.6–2.7)	1.1 (0.6–1.6)	0.8 (<0.6–1.3)	0.008 (<0.006–0.014)
Lettuces (n:25)	Fields (n:8)	6.5 (5.7–7.3)	0.6 (<0.6 - <0.6)	2.0 (<0.6–3.6)	0.6(<0.6–1.5)	0.3(<0.6–1.6)	0.078 (<0.006–0.202)
	Marketplace (n:8)	6.3 (5.3–7.3)	0.8 (<0.6–6.0)	1.9 (<0.6–5.1)	1.1 (0.6–1.8)	0.7(<0.6–1.3)	0.07(<0.006–0.141)
	Supermarket (n:9)	5.8(4.7–6.7)	<0.6(<0.6 - <0.6)	1.2 (<0.6–3.3)	1.1(0.6–1.7)	0.7(<0.6–2.2)	0.101(<0.006–0.271)

**():** Minimum - Maximum, **TC**: Total Coliform**, SRC**: Sulphite Reducing Clostridia, **SSRC**: Spores of Sulphite-Reducing Clostridia, **NMP**: Most Probable Number, **CFU**: Colony Unit Former y <: Limited de detection.

The presence of *Clostridium* sp. in both vegetative and spore forms (SSRC) was observed at a higher percentage compared to other bacterial indicators evaluated, except for total coliforms. The presence of both forms of *Clostridium* sp. was found in a range from 37.5 % (3/8) to 88.9 % (8/9) (Fig. 2). The difference in mean concentrations between the vegetative and spore forms did not exceed 0.4 Log_10_ CFU/g (Table 1).

In lettuce samples, total coliforms were obtained in all samples analyzed, while *E. coli* was only detected in one sample obtained from a marketplace, with a maximum concentration of 6.0 (Log_10_ CFU/g) (Table 1). On the other hand, Enterococcus was detected more frequently in both field and marketplace samples (62.5 %; 5/8), followed by supermarkets (44.4 %; 4/9) (Fig. 2). However, the highest mean concentration was observed in both field and marketplace samples, with the maximum value corresponding to samples obtained from marketplaces (5.1 Log_10_ CFU/g) compared to the mean and maximum values of samples acquired from supermarkets (Table 1).

The presence of *Clostridium* sp. sulfite-reducing both in its vegetative (SRC) and spore (SSRC) forms was detected in samples from different origins, observing a higher presence in its vegetative form in samples from marketplaces and chain markets, whereas the presence of the spore form (SSRC) decreased to an incidence between 62.5 % (5/8) and 55.5 % (5/9) (Fig. 2). In field samples, an incidence of *Clostridium* sp. was mainly observed in its vegetative form (50 %; 4/8) compared to the spore form (25 %; 2/8) (Fig. 2).

The presence of *Salmonella* spp. was reported more frequently in lettuce samples (77.7 %–87.5 %) compared to strawberry samples (Fig. 2), showing a higher variation in incidence depending on the places of origin, such as marketplaces (62.5 %; 5/8), supermarkets (33.3 %; 3/9), and fields (12.5 %; 1/8), contrasting with what was observed in lettuce samples where the percentages of presence among different origins are close to each other (77.5 % and 87.5 %; 7/9 and 7/8) (Fig. 2).

### 3.2 Viral indicators in strawberries and lettuces

**Fig. 3:**
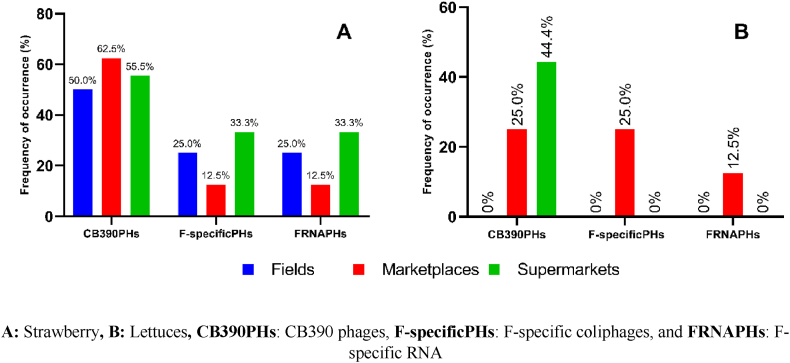
**Frequency of appearance of viral indicators (phages) in strawberry and lettuce samples**. **A:** Strawberry**, B:** Lettuces**, CB390PHs**: CB390 phages, **F-specificPHs**: F-specific coliphages, and **FRNAPHs**: F-specific RNA.

Phages of CB390 were detected in strawberries from markets (62.5 %; 5/8), supermarkets (55.5 %; 5/9), and fields (50 %; 4/8), whereas the detection of Specific Phages - RNA was lower, as observed in samples from supermarkets (33.3 %; 3/9), fields (25 %; 2/8), and markets (12.5 %; 1/8) (Fig. 3). The maximum average concentration of CB390 Phages Log_10_ PFU/50 g was observed in samples from fields (4.6 Log_10_ PFU/50 g), compared to those reported in supermarket (3.5 Log_10_ PFU/50 g) and market samples (4.2 Log_10_ PFU/50 g). Regarding Specific Phages - RNA, similar concentrations were found (2.9–3.3 Log_10_ PFU/50 g), with no significant differences among samples from the three different sources (Table 2).

**Tabla 2:** Concentrations of viral indicators in the different samples of strawberries and lettuce.

Matrix	Origin	CB390PHsLog_10_(UFP/50 g)	F-specificPHs Log_10_(UFP/50 g)	FRNAPHsLog_10_(UFP/50 g)
Strawberry (n:25)	Fields (n:8)	4,6(<0,8–3,3)	3,1 (<0,8–3,3)	3,1 (<0,8–3,3)
	Marketplace (n:8)	4,2(<0,8–3,8)	3.3 (<0,8–3,6)	3.3 (<0,8–3,6)
	Supermarket (n:9)	3.5 (<0,8–4,3)	2,9 (<0,8–4,2)	2,9 (<0,8–4,2)
Lettuces (n:25)	Fields (n:8)	<0,8 (<0,8 - <0,8)	<0,8 (<0,8 - <0,8)	<0,8 (<0,8 - <0,8)
	Marketplace (n:8)	1,3 (<0,8–1,7)	0,9 (<0,8–2,4)	0,7 (<0,8–0,8)
	Supermarket (n:9)	1,2 (<0,8–2)	<0,8 (<0,8 - <0,8)	<0,8 (<0,8 - <0,8)

**():** Minimum - Maximum, **CB390PHs**: CB390 phages, **F-specificPHs**: F-specific coliphages, **FRNAPHs**: F-specific RNA coliphages, **UFP**: Plaque Forming Units, and (**<**): Limit of detection.

In the case of lettuce samples from fields, the presence of CB390 Phages and Specific Phages - RNA was not detected (Fig. 3), possibly because the samples were collected during the rainy season, meaning that water sources were not used for irrigation. Concerning samples acquired from markets, a prevalence of 25 % (2/8) and 12.5 % (1/8) was found for CB390 and Specific Phages - RNA, respectively, while only CB390 Phages were detected in supermarket samples (44.4 %; 4/9) (Fig. 3).

### 3.3 Molecular markers for discriminating the origin of fecal contamination and *H. pylori* in strawberries and lettuce

**Fig. 4:**
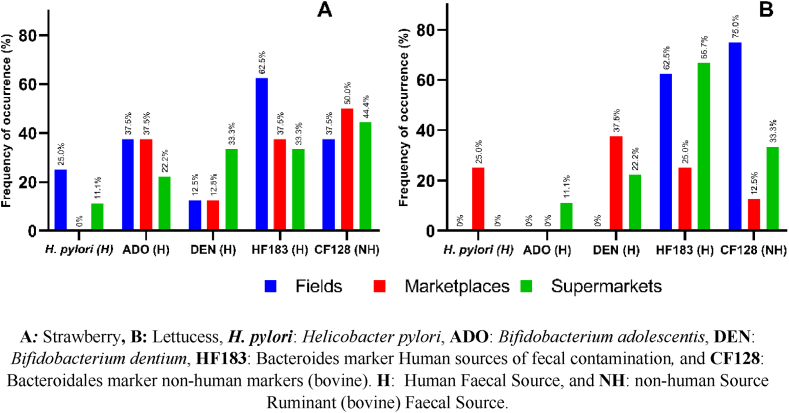
Frequency of appearance of molecular markers and *H. pylori* in strawberry, and lettuce samples. **A*:*** Strawberry**, B:** Lettucess, ***H. pylori***: *Helicobacter pylori*, **ADO**: *Bifidobacterium* adolescentis, **DEN**: *Bifidobacterium dentium*, **HF183**: Bacteroides marker Human sources of fecal contamination*,* and **CF128**: Bacteroidales marker non-human markers (bovine). **H**: Human Faecal Source, and **NH**: non-human Source Ruminant (bovine) Faecal Source.

The animal-origin *Bacteroides* marker (CF128) was primarily found in strawberry samples from marketplaces (50 %; 4/8), followed by supermarkets (44.4 %; 4/9) and fields (37.5 %; 3/8) (Fig. 4). Conversely, in lettuce samples testing positive, the marker's presence was higher in those originating from fields (75 %; 6/8), compared to 33.3 % (3/9) from supermarkets and 12.5 % (1/8) from marketplaces. Regarding the human-origin marker (HF183), it had a higher incidence in strawberry samples from fields (62.5 %; 5/8) compared to marketplaces (37.5 %; 3/8) and supermarkets (33.3 %; 3/9). In lettuce samples, the marker showed similar percentages between those from supermarkets (66.7 %; 6/9) and fields (62.5 %; 5/8), followed by marketplaces (25 %; 2/8) (Fig. 4).

Regarding the *Bif. adolescentis* (ADO) markers, they were detected in a higher percentage compared to DEN (*B. dentium*). Specifically, in strawberry samples from fields and marketplaces, the ADO marker was detected in 37.5 % (3/8) of them, while DEN was detected in 12.5 % (1/8) in both origins and in 33.3 % (3/9) of samples from chain markets, compared to 22.2 % (2/9) of ADO (Fig. 4). In lettuce samples, the ADO marker was only detected in those from supermarkets (11.1 %; 1/9), while DEN was detected in 22.2 % (2/9) of samples obtained from markets and in 37.5 % (3/8) of those acquired from marketplaces (Fig. 4).

*H. pylori* was detected in strawberry samples from fields at 25 % (2/8) and those obtained from supermarkets at 11.1 % (1/9), while in lettuce samples, it was detected in those from marketplaces at 25 % (2/8) (Fig. 4).

## 4 Discussion

### 4.1 Total coliforms

The detection of coliforms in all strawberry and lettuce samples from the three origins highlights the lack of microbiological quality control in the production chain of these foods. However, it cannot be asserted that the presence of this indicator is due to fecal contamination since its origin is nonspecific [50]. Nevertheless, the presence of total coliforms in the analyzed products leads to a decrease in their shelf life, causing economic losses. It was observed that the coliform concentrations obtained in this study were higher, with an equal positivity rate (100 %), compared to those reported in the same types of foods in countries like Egypt [13]. Similarly, the incidence rate and concentrations in strawberries are higher than those reported in Spain and higher than those reported in lettuce in Sweden. It is estimated that the high coliform concentrations are due to the poor quality of water used for agricultural irrigation in the study area compared to the water quality in the mentioned countries, which is of superior quality.

It is noteworthy that all samples analyzed in this study, regardless of their origin, presented coliforms at concentrations ranging from 5.3 to 6.5 (Log_10_ CFU/g). These results draw attention to samples from supermarkets (Table 1 y Fig. 2), as these establishments demand compliance with *Good Agricultural Practices* (GAP) and *Good Manufacturing Practices* (GMP), as well as a certain degree of food quality, in addition to providing training on food handling. This differs from the handling of such foods from the fields and marketplaces in Colombia. Despite this, other studies have shown a marked differentiation in the low concentrations of total coliforms detected in supermarkets compared to samples from cultivation fields [51] and local markets [52], where higher counts are observed.

### 4.2 Escherichia coli

Regarding the presence of *E. coli* in one sample of strawberries from fields, one from marketplaces, and one in lettuce from a marketplace (Table 1 and Fig. 2), the maximum levels are higher than those found in countries like the United States [53], Sweden [16], and Egypt [13], but with a lower prevalence compared to that reported in Egypt (58 %–73 %) [13] and Sweden (66.6 %) [16], as opposed to the findings in this study (13 %); however, the results obtained also differ from other studies reporting lower counts and prevalence [[53], [54], [55], [56]]. The quantification of *E. coli* in strawberries and lettuce from the mentioned sources allows for the evidence that they do not comply with the standards established in the current regulations in Colombia (Resolution No. 1407 of 2022) [57], which sets the microbiological criteria for foods and beverages intended for human consumption, in this case for fresh, peeled, and/or cut fruits and vegetables, which was set at 10 CFU/g. On the other hand, it was evidenced that samples obtained in supermarkets did not present *E. coli* (Table 1 and Fig. 2), which coincides with what was reported in Spain by Ortiz-Sóla et al., 2020 [51], for these cases, it is inferred that the absence of the bacteria is due to cleaning and disinfection processes carried out prior to sale to the end consumer.

### 4.3 *Clostridium* and sulfite-reducing spores (SRC/SSRC)

The prevalence of *Clostridium* sp. sulfite-reducing both in its vegetative and spore forms in strawberries (38–89 %) and lettuce (25–100 %), and the concentrations found were very similar regardless of the sample's origin (Table 1). These findings differ from the concentration and prevalence of *Clostridium* spp. reported in crops by Oliveira et al., 2019 [56], where they obtained a prevalence of 3.3 % in strawberries, and by Ahlinder et al., 2022 [16] al, with maximum counts of 10 CFU/g in lettuce. The presence of *Clostridium* spp. in both types of samples may be due to this microorganism being widely distributed in the environment, also found in soil. Therefore, during the planting and harvesting stages of the food, they may come into contact with this bacterium [58]. However, it is noteworthy the minimal difference between the counts of the vegetative form and the spores of *Clostridium* spp. (≤0.4 Log_10_ CFU/g), since whether or not any disinfection process is carried out on these types of foods, the levels will remain significant due to the resistance presented by the spores [59], becoming a problem for entities related to food control and public health, due to the implications they can generate.

### 4.4 Enterococcus

The levels of Enterococcus found in strawberries and lettuce are noteworthy due to the similar concentrations between the same types of food. Additionally, the prevalence of this bacterium in strawberries (25–44.4 %) and lettuce (44.4–62.5 %) is higher than that of *E. coli* (≤12.5 %) (Table 1 and Fig. 2). These results contrast with another study in which Enterococcus was not detected in lettuce crops [16]. It is worth noting that *Enterococcus* spp. is resistant to changes in temperature, pH, and dehydration [60], leading to its persistence over time when disinfection treatments are inadequate or not performed according to established protocols [[61], [62], [63]], remaining in the food until consumption.

### 4.5 *Salmonella* spp.

A difference in the incidence of *Salmonella* spp. was observed between strawberries (12.5%–62.5 %) and lettuce (77.7%–87.5 %) (Fig. 2), as well as in the maximum concentrations found (Table 1), which is in stark contrast to the findings reported by Ortiz-Solà et al., 2020 [51], Ahlinder et al., 2022 [16], and Oliveira et al. (2019) [56], where the presence of *Salmonella* spp. was not evident. However, similarities were found with studies conducted in Egypt [13] and Bogotá (Colombia), where the presence of this pathogen was reported in samples from crops [64,65] and obtained from marketplaces [66]. The presence of *Salmonella* spp. in both types of food and in all evaluated sources highlights that these foods do not comply with the regulations established in Colombia (Resolution No. 1407 of 2022) [57], which stipulates that this bacterium should not be found in fresh, peeled, and/or cut fruits and vegetables, setting the standard at Absence/25 g. With regards to the presence of *Salmonella* spp., the serious implications are underscored, such as its ability to resist or persist in different types of environments and cause salmonellosis, one of the most significant foodborne illnesses leading to severe complications in both humans and animals [67,68].

### 4.6 CB390 phages and F-specific RNA phages

The detection of phages in strawberries and lettuce is of great importance as they are considered and used as indicators of the presence of enteric viruses. Therefore, their detection raises concerns about the health implications of consuming foods contaminated with these viruses [53,[69], [70], [71]]. Regarding viral indicators in strawberries, the presence of CB390 phages was detected in over 50 % of the samples, while F-specific RNA phages were found in samples from supermarkets (33.3 %), farms (25 %), and markets (12.5 %) (Fig. 3). CB390 phages exhibit higher incidence compared to FRNAPHs, attributed to the ability of the *E. coli* CB390 strain to capture two groups of phages [44]. Somatic phages are more widely distributed in the environment [72] but are less resistant to disinfectants and UV light [73], unlike FRNAPHs [74,75]. Therefore, the significance of the latter lies in their utility as indicators for food processing and treatment processes [[76], [77], [78]].

The results obtained in lettuce from farms contrast with those reported in studies conducted by Yazdi et al., 2017 [71], and Shin et al., 2019 [79], where the presence of F-RNA phages was reported in 25 %–80 % and 13.3 % of the samples analyzed from farms, respectively. Additionally, the concentrations of CB390 phages in lettuce from supermarkets and markets, and F-RNA phages in samples from markets, differ from those reported by Tsuei et al., 2007 [80] where no coliphages were detected in commercially available vegetables. Similarly, a study conducted in the United States [53] reported the presence of specific RNA phages in lettuce sales, with 47 % of the processed or handled sample group showing a higher incidence of specific RNA phages compared to those that were not processed (19 %) [53].

### 4.7 Helicobacter pylori

The presence of *H. pylori* DNA in some samples of strawberries from farms (25 %) and supermarkets (11.1 %), as well as in lettuce acquired from markets (25 %), coincides with studies where *H. pylori* DNA was detected in 20 % [81] to 83.3 % [82] of unwashed or fresh lettuce samples, and in 14 %–35 % of other types of unwashed or fresh vegetables or salads [81,83].

The presence and detection of *H. pylori* DNA in the analyzed samples indicate strictly human fecal contamination, as this bacterium is species-specific and found only in the human gastrointestinal tract [84]. However, it is important to note that the presence of this DNA in food does not pose a risk to the consumer, as its presence does not imply the viability of the bacterium and therefore a direct source of infection. On the other hand, its use as a possible complementary or differentiating marker of human fecal contamination should continue to be investigated and evaluated, as the presence of this bacterium in different matrices will occur whenever it is present in the stomach and excreted in feces. In this regard, there is a high probability of this process occurring, given that *H. pylori* prevalence in Latin America ranges from 70 % to 90 % [85] and in Colombia from 77.2 % to 83 % [[86], [87], [88]].

However, the detection of this bacterium's DNA in food presents some limitations, as the genetic material may degrade during the food production chain due to variables such as exposure to high temperatures, sunlight, humidity, and the presence of different agrochemicals and cleaning and disinfection products, which can affect the integrity of the genetic material [89,90].

### 4.8 *Bacteroides* (HF183 and CF128) and *Bifidobacterium* (ADO and DEN) markers

The detection of *Bacteroides* markers differentiating between human (HF183) or non-human (bovine/CF128) fecal contamination in samples of strawberries and lettuce (Fig. 2) reveals mixed contamination in both types of foods regardless of their origin. A higher presence of the human origin marker (HF183) was observed in strawberries (62.5 %) compared to the bovine marker (CF128) (50 %); the opposite was observed in lettuce samples (CF128: 75 % and HF183: 66.7 %) (Fig. 2). The literature reports the presence of *Bacteroides* in different types of foods using non-origin differentiating markers of fecal contamination [30,91]. On the other hand, Ravaliya et al., 2014 [92] reported that 39 % of samples of tomatoes, jalapeño peppers, and melons had *Bacteroides*, with 46 % showing human contamination and none showing bovine contamination. The results confirm that the detection of *Bacteroides* markers in food is stable and resistant over time compared to other fecal contamination indicators [30], allowing for evaluation throughout the food production chain, making them a promising marker due to their direct relationship with the origin of human or non-human (animal) fecal contamination [46].

Furthermore, the presence of *B. adolescentis* (ADO) and *B. dentium* (DEN) in strawberries was observed in all evaluated origins, with a higher detection of ADO in samples from cultivation and obtained in marketplaces (Fig. 4). In lettuce, ADO and DEN were detected only in samples acquired from supermarkets, and DEN exclusively in those from marketplaces, with the latter showing a higher incidence in both origins. The use of these markers ensures that the fecal contamination present is strictly human [[93], [94], [95]], despite differences in specificity and sensitivity reported between these two markers [93,96,97].

The presence of microbiological indicators such as *E. coli* and Enterococcus, *C. perfringens* spores and vegetative form, coliphages, *Salmonella* spp., and molecular markers for differentiating the source of fecal contamination (HF187, CF128, ADO, and DEN), and *H. pylori* DNA in the different samples analyzed (Fig. 2, Fig. 3, Fig. 4 and Table 1, Tabla 2), in addition to indicating deficiencies in the food production chain [98,99], pose a significant risk to food safety and consumer health, as these foods are consumed directly or mixed in salads. Therefore, strict control and monitoring by the entities responsible for food production control and health authorities are necessary. In Latin America, approximately 77 million people get sick each year from contaminated food, with bacteria (69 %), chemicals (19.5 %), viruses (9.7 %), and parasites (1.8 %) being the most common contaminants [100]. In Colombia, from 2011 to 2021, 8955 outbreaks have been reported, with an average of 814 outbreaks per year [100].

Moreover, the use of surface water unsuitable for agricultural irrigation increases the risk of microbiological contamination of fecal origin in food, such as the water from the Bogotá River (Fig. 1A), which presents significant concentrations of traditional indicators (bacteria and phages) [[101], [102], [103]], pathogenic microorganisms [104], clear and persistent human and animal contamination [102,103], and also the presence of *H. pylori* DNA [34,35], making it one of the main sources of contamination for these types of foods due to deficiencies in handling, storage, distribution, among others.

Another factor to highlight is the presence of the microorganisms and markers evaluated in foods from marketplaces and supermarkets (Fig. 2, Fig. 3, Fig. 4 and Table 1, Tabla 2), despite these places currently having more controls by health authorities, as well as the obligation to implement and certify Good Manufacturing Practices (GMP) for product sales [105], and sometimes Good Agricultural Practices (GAP) for product sales to supermarkets or large retailers.

Furthermore, the risk to consumers would increase even more due to the increasing preference for buying food in supermarkets (85 %), as there is a perception that hygiene conditions in these places are high, leaving behind neighborhood stores (13 %) and marketplaces (2 %), which are perceived inaccurately in most cases [106]. Detecting viral markers and indicators in various sample types informs regulatory bodies about the imperative to strengthen current regulations and explore the integration of new markers and indicators. This effort is crucial for safeguarding the safety of products intended for direct consumption.

## 5 Conclusions

The present study is the first to collectively determine the presence of traditional indicators, viral indicators, and molecular markers of fecal contamination and *H. pylori* in raw food products, specifically strawberries and lettuce in Colombia. Overall, a mixed fecal contamination was found in samples from all three evaluated sources, with the presence of markers indicating both human and bovine fecal contamination. The detection of *H. pylori* genetic material in the analyzed food samples could be proposed as a potential molecular marker to be included in the group of Microbial Source Tracking indicators. Additionally, it is important to note that the presence of such genetic material does not represent a proven risk to consumers, thus further analyses are suggested to determine the viability of the bacteria and whether the food serves as a vehicle for consumer infection.

Most of the analyzed strawberry and lettuce samples comply with the permissible limits of bacteria indicated in Colombian regulations [57], such as the *E. coli* count, except for the presence of *Salmonella* spp. The other bacterial, viral, and molecular markers are not regulated, but their detection, evaluation, and monitoring are still relevant.

The presence of fecal contamination in the analyzed samples has significant implications for food safety, confirming the need to implement preventive and control measures throughout the production chain to ensure the safety of these products before distribution to the final consumer.

In general, consumers are encouraged to take appropriate hygiene measures when handling and preparing food, especially raw foods. Likewise, health authorities and regulatory bodies are urged to strengthen surveillance processes for food quality, while producers and marketers are encouraged to improve production and handling practices to ensure food safety.

The authors aim to include and identify a greater number of MST markers and pathogenic microorganisms in foods intended for direct consumption from various regions of the country.

## Data availability statement

Data Will not be made available.

## CRediT authorship contribution statement

**Fidson-Juarismy Vesga:** Writing – review & editing, Writing – original draft, Supervision, Project administration, Methodology, Investigation, Formal analysis, Data curation, Conceptualization. **Camilo Venegas:** Writing – review & editing, Writing – original draft, Methodology, Formal analysis, Data curation. **Valentina Flórez Martinez:** Writing – original draft, Investigation, Formal analysis. **Andrea C. Sánchez-Alfonso:** Writing – original draft. **Alba Alicia Trespalacios:** Writing – review & editing, Writing – original draft, Supervision, Resources, Project administration, Funding acquisition, Conceptualization.

## Declaration of competing interest

The authors declare the following financial interests/personal relationships which may be considered as potential competing interests: Alba Alicia Trespalacios Rangel reports was provided by Ministerio de Ciencias, Tecnología e Innovación. If there are other authors, they declare that they have no known competing financial interests or personal relationships that could have appeared to influence the work reported in this paper.
